# Machine‐learning classification of bipolar disorder incorporating wearable‐derived core body temperature and actigraphy‐derived sleep indices

**DOI:** 10.1002/pcn5.70359

**Published:** 2026-06-08

**Authors:** Kazuhiro Kurihara, Ayano Shiroma, Masaki Kamata, Naruyo Yoshimoto, Ayano Saso, Akiyoshi Shimura, Yukiko Imai, Tadashi Miyahara, Yoshikazu Takaesu

**Affiliations:** ^1^ Department of Neuropsychiatry, Graduate School of Medicine University of the Ryukyus Ginowan Okinawa Japan; ^2^ Department of Somnology Tokyo Medical University Shinjuku‐ku Tokyo Japan; ^3^ ACCELStars Inc. Bunkyo‐ku Tokyo Japan

**Keywords:** actigraphy, bipolar disorder, circadian rhythm, core body temperature, phase angle difference

## Abstract

**Aim:**

This study aimed to classify patients with bipolar disorder (BD) and normal controls (NCs) using machine‐learning models that incorporate wearable‐derived core body temperature (CBT) and actigraphy‐derived sleep indices.

**Methods:**

We enrolled 23 euthymic patients with BD and 43 NCs. Sleep parameters were collected via wrist actigraphy for 14 days, and CBT was measured with a wearable device for 3 days to estimate the CBT nadir and its phase differences with sleep indices. Models were constructed with a base model using CBT‐ and sleep‐derived features, and an extended model that included sociodemographic variables. Features were standardized (StandardScaler), and classifiers (random forest, LightGBM, and XGBoost) were evaluated. Hyperparameters were optimized using cross‐validation within the training data. Performance was evaluated using repeated nested cross‐validation. SHapley Additive exPlanations (SHAP) values were computed in the extended model to quantify relative feature contributions to the model output.

**Results:**

In nested cross‐validation at the MaxF1 operating point, the mean area under the receiver operating characteristic curve (ROC–AUC) was 0.771 ± 0.162 for the base model and 0.930 ± 0.050 for the extended model. In the extended model, SHAP suggested that total sleep time, wake time, and the wake time–CBT nadir phase difference were features potentially associated with the model output.

**Conclusion:**

In this small‐sample study, a classification approach combining wearable‐derived CBT indices and actigraphy‐based sleep parameters suggested preliminary discrimination between BD and NC. Further validation is warranted in larger cohorts with balanced background characteristics, including disorders requiring differential diagnosis.

## Graphical abstract

A machine‐learning approach combining wearable‐derived core body temperature (CBT) and actigraphy‐derived sleep indices showed preliminary discrimination between euthymic patients with bipolar disorder (BD) and normal controls. Total sleep time, wake time, and the wake time–CBT nadir phase difference were key contributors.

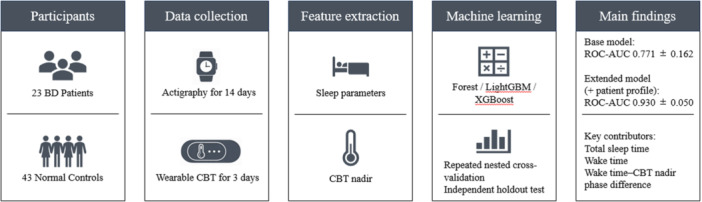

## INTRODUCTION

Bipolar disorder (BD) is a recurrent mood disorder characterized by alternating episodes of mood elevation and depression.^1^, ^2^, ^3^ In clinical practice, BD symptoms often fluctuate over time, highlighting the need for continuous and objective monitoring. In this context, wearable devices have recently attracted attention as tools for objective and continuous assessment.^4^, ^5^, ^6^, ^7^ Previous studies have shown that disturbances in sleep–wake rhythm, as measured by actigraphy, are associated with functional impairment in patients with BD.^8^ Sleep–wake rhythm parameters are also related to brain functions involved in working memory.^9^ Furthermore, delayed sleep–wake rhythms predict functional impairments.^10^ These findings suggest that wearable devices may help capture clinically important aspects of BD.

Wearable devices have also been increasingly recognized as valuable tools for machine‐learning–based classification of BD. Machine‐learning approaches using actigraphy data have successfully distinguished patients with BD and normal controls (NCs) based on long‐term motor activity,^11^ combined activity, and sleep variability parameters.^12^ Similar actigraphy‐based approaches have been applied to pediatric and adolescent populations with BD.^13^, ^14^ Furthermore, deep learning models integrating demographic and lifestyle information with actigraphy‐derived activity data have been explored.^15^ These findings highlight the potential of wearable devices in machine‐learning approaches for BD.

In recent years, wearable devices capable of estimating core body temperature (CBT) from skin temperature have gained increasing research interest. CBT has long been recognized as a valid circadian phase marker and a key physiological indicator, alongside melatonin and cortisol.^16^ Traditionally, CBT has been measured via rectal temperature; however, this method has limited practicality owing to the nature of the insertion site.^17^ To address this, wearable devices that noninvasively estimate CBT have been developed. Their validity has been examined against rectal temperature,^18^ and several studies have employed such sensors in various research settings.^19^, ^20^

However, wearable‐derived CBT has rarely been used in BD research to date. The conventional method for measuring CBT via rectal temperature is limited by impracticality in research settings. A previous study reported a misalignment between the CBT nadir and sleep phase in patients with delayed sleep phase syndrome (DSPS),^21^ whereas studies targeting patients with BD remain scarce. In addition, another study reported an association between BD and circadian rhythm disturbances, particularly delayed sleep–wake rhythms.^22^ Considering this association, wearable‐derived CBT, which can be applied more easily than conventional methods, may help elucidate the characteristics of BD.

Thus, this study aimed to evaluate whether BD and NC can be classified using machine‐learning models that integrate wearable‐derived CBT indices and actigraphy‐derived sleep parameters. This work was designed as an exploratory, proof‐of‐concept analysis in a small‐sample setting, representing a step toward the development of diagnostic support tools that leverage wearable‐derived circadian measures.

## METHODS

### Participants

We enrolled outpatients with BD, diagnosed based on the Diagnostic and Statistical Manual of Mental Disorders, Fifth Edition,^23^ who were in a euthymic state and attending the University of the Ryukyus Hospital between October 4, 2023, and August 31, 2025. NCs with no history of psychiatric disorders or treatment were recruited during the same period. Inclusion criteria common to both groups required participants to be aged 18–65 years.

Exclusion criteria included shift work within the past 3 months, comorbid primary sleep disorders (including sleep apnea and restless legs syndrome), alcohol or drug dependence, dementia, severe physical illness, and the presence of implanted electronic medical devices in the participants or their cohabiting family members (for safety considerations and to avoid potential interference with wearable sensor signals). Additional exclusion criteria for patients with BD included being within 1 month of remission, having visual or language impairments, an estimated premorbid intelligence quotient below 80, and acute suicidal ideation. Healthy controls were excluded if they had any psychiatric history or were undergoing treatment. After applying these criteria, 23 patients with BD and 43 healthy controls were included in the final analysis.

### Assessments

Demographic information, including age, sex, years of education, marital status, living arrangements (living alone or not), family history of psychiatric disorders, and employment status, was obtained from all participants. For patients with BD, additional clinical information was collected, including BD subtype (Type I or II), age at onset, illness duration, current mood symptom severity assessed by the 17‐item Hamilton Depression Rating Scale (HAMD‐17) and the Young Mania Rating Scale (YMRS), and medication status. Medication use was recorded for mood stabilizers, antipsychotics, hypnotics, and antidepressants, as well as the specific agents prescribed at the time of assessment.

Subjective sleep problems were assessed using the Japanese version of the Insomnia Severity Index (ISI), a validated 7‐item questionnaire frequently used to screen the severity of insomnia.^24^, ^25^ Each item is rated on a 5‐point Likert scale (0–4) and covers aspects such as difficulty initiating sleep, maintaining sleep, satisfaction with sleep, and the impact of insomnia on daily functioning. The total score ranges from 0 to 28, with higher scores indicating more severe insomnia.

Functional disability was evaluated using the 12‐item version of the World Health Organization (WHO) Disability Assessment Schedule 2.0 (WHODAS 2.0).^26^ This standardized self‐report instrument, developed by the WHO, assesses health‐related challenges across six domains: cognition, mobility, self‐care, interpersonal relationships, life activities, and social participation. Each item is scored on a 5‐point scale from 1 (none) to 5 (extreme or cannot do), with higher total scores reflecting greater impairment.

Depressive symptoms were assessed using the HAMD‐17,^27^, ^28^ a clinician‐administered instrument widely used in both clinical practice and research. Each item is scored on anchored 3‐ or 5‐point scales (0–2 or 0–4) and evaluates core domains such as mood, sleep disturbance, psychomotor changes, anxiety, and somatic symptoms. Total scores range from 0 to 52, with higher scores indicating greater severity of depressive symptoms.

The severity of manic symptoms was assessed using the YMRS,^29^ which consists of 11 items, with four items rated from 0 to 8 and the remaining seven rated from 0 to 4. The total score ranges from 0 to 60, with higher scores indicating more severe manic symptoms.

**Figure 1:**
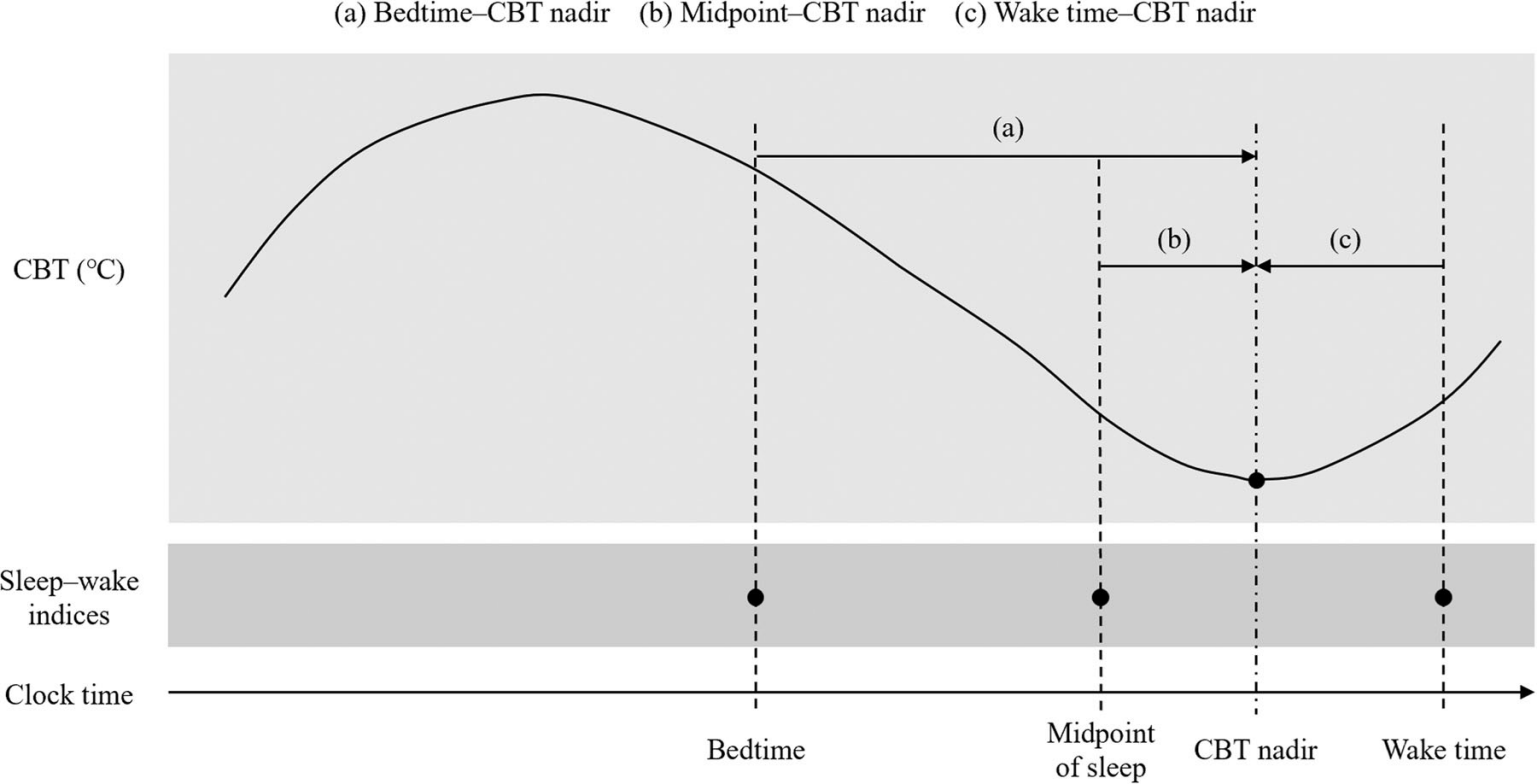
Conceptual illustration of phase differences between bedtime, sleep midpoint, wake time, and the core body temperature (CBT) nadir. *Note*: In this study, phase‐difference indicators were calculated relative to the CBT nadir time as follows: bedtime–CBT nadir phase difference (min) = CBT nadir time − bedtime. Midpoint–CBT nadir phase difference (min) = CBT nadir time − sleep midpoint. Wake time–CBT nadir phase difference (min) = wake time − CBT nadir time.

CBT was continuously monitored using a CORE sensor (greenTEG AG, Zurich, Switzerland; distributed in Japan by corebodytemp.jp) for 3 consecutive days. The CBT nadir phase (o'clock) was estimated using cosinor analysis based on the 3‐day dataset. Subsequently, phase differences were calculated relative to the CBT nadir time as follows: The bedtime–CBT nadir phase difference was defined as CBT nadir time − bedtime; the midpoint–CBT nadir phase difference as CBT nadir time − sleep midpoint; and the wake time–CBT nadir phase difference as wake time − CBT nadir time (Figure 1). Previous studies have demonstrated the acceptable validity of the CORE sensor under static environmental conditions.^18^

Objective sleep–wake patterns were assessed using wrist‐worn actigraphy (ACCEL POLARIS; ACCELStars Inc., Tokyo, Japan), which employs a jerk‐based algorithm for sleep–wake classification based on triaxial acceleration data. The validity of the ACCEL algorithm has been demonstrated against polysomnography, showing >90% sensitivity and >80% specificity for sleep–wake discrimination.^30^ Actigraphy‐derived indices included total sleep time (min), bedtime (o'clock), midpoint of sleep (o'clock), wake‐up time (o'clock), sleep efficiency (%), wake after sleep onset (min), and standard deviations (SDs) of bedtime, midpoint of sleep, and wake‐up time across the recording period. For each participant, mean values across the 14‐day recording were used for analysis, and variability measures (SDs) were calculated across the same period.

All questionnaires were administered on the first day of the study. The ISI and WHODAS 2.0 were administered to both the BD and NC groups, whereas HAMD‐17 and YMRS were administered to only the BD group. In addition, actigraphy and CBT measurements were conducted in both groups for 14 and 3 consecutive days, respectively, following study enrollment.

### Statistical analyses

Group comparisons of demographic characteristics and wearable device‐derived indices were performed using the Mann–Whitney *U* test for continuous variables. For categorical variables, Pearson's chi‐square or Fisher's exact test was applied, depending on the expected cell counts. To account for multiple comparisons among CBT‐ and actigraphy‐derived indices, false discovery rate (FDR) correction was applied using the Benjamini–Hochberg method.

During the 3‐day CBT recording period, data‐quality metrics were assessed to evaluate measurement stability. Specifically, wearable recording time, missingness rate, and adherence were calculated for each participant. Overall cosinor model fit for CBT rhythm estimation was summarized using the coefficient of determination (*R* ^2^). The mean CBT nadir time was computed using both all available CBT segments and those restricted to segments demonstrating moderate model fit (*R* ^2^ > 0.3). The difference between these estimates was calculated to evaluate the impact of model‐fit restriction. In addition, day‐to‐day within‐subject variability of the estimated CBT nadir timing across the 3‐day period was quantified using the within‐subject variance.

To examine whether CBT and sleep indices were independently associated with diagnostic group after accounting for the potential influence of sociodemographic factors that may reflect social schedule and lifestyle/occupational structure, multivariable linear regression analyses were conducted. Each CBT nadir indicator and actigraphy‐derived sleep parameter was entered as a dependent variable, while diagnostic group (BD = 1) together with sociodemographic covariates (age, sex, years of education, marital status, living alone, and employment status) were included as independent variables. Unstandardized regression coefficients (*B*) and 95% confidence intervals (CIs) were estimated.

All statistical tests were two‐tailed, and *p* < 0.05 was considered statistically significant. All analyses were performed using IBM spss Statistics for Windows, v28.0 (IBM Corp., Armonk, NY, USA).

Machine‐learning analyses were conducted using a nested cross‐validation framework to obtain robust and unbiased performance estimates in this small‐sample dataset. First, the dataset was randomly divided into a training set (*n* = 52) and an independent holdout test set (*n* = 14) using stratified sampling to preserve class proportions. The holdout set was not used during model selection or hyperparameter optimization; rather, it was used only as a supplementary assessment. Within the training set, model selection and hyperparameter optimization were performed using repeated stratified fivefold cross‐validation in the inner loop (5 folds × 3 repeats), implemented via GridSearchCV. Candidate classifiers included random forest, LightGBM, and XGBoost. Hyperparameters were optimized by maximizing the mean cross‐validated area under the receiver operating characteristic curve (ROC–AUC). Feature preprocessing included standardization using StandardScaler. Principal component analysis (PCA) was not applied in the final analyses in order to retain interpretability in the original feature space. To assess the stability and generalizability of the model selection procedure, nested cross‐validation was implemented within the training data using an outer loop of stratified fivefold cross‐validation repeated five times (5 folds × 5 repeats). For each outer fold, hyperparameter optimization was performed exclusively within the corresponding training partition, and performance was evaluated on the held‐out outer fold. The primary performance evaluation was based on repeated nested cross‐validation within the training data. Performance metrics were summarized across the outer folds and reported as the mean, SD, and 95% CIs derived from the empirical outer‐fold distribution. For the supplementary assessment, the selected modeling pipeline was refitted on the entire training dataset (*n* = 52), and the model was evaluated on the independent holdout test set (*n* = 14).

To evaluate the potential impact of sociodemographic factors that may reflect social schedule or lifestyle/occupational structure, two feature sets were examined. The base model included only CBT‐related parameters and actigraphy‐derived sleep indices. An extended model additionally incorporated age, sex, years of education, marital status, living arrangement, and employment status as input features. Model performance was compared between these configurations to assess whether classification results were influenced by background sociodemographic characteristics.

Multiple operating points were examined. The primary classification threshold was defined as the threshold that maximized the F1 score (F1‐max) based on out‐of‐fold predicted probabilities within the training data. In addition, supplementary operating points prioritizing sensitivity (targeting sensitivity ≥ 0.90) and specificity (targeting specificity ≥ 0.90) were evaluated. For the sensitivity‐ and specificity‐prioritized operating points, among thresholds meeting the target criterion, we selected the threshold that maximized the complementary metric (specificity or sensitivity, respectively). All thresholds were determined using out‐of‐fold predicted probabilities within the training data. Performance at each operating point was evaluated on the outer test folds and summarized as mean ± SD across folds.

Model interpretability was evaluated using SHAP as a measure of the relative contributions (feature importance) of each feature to the model output. SHAP values were computed for the final fitted extended model (sleep + CBT + sociodemographic variables), allowing feature contributions to be evaluated while accounting for the sociodemographic variables included in the model. SHAP values were calculated using the standardized original features (after applying StandardScaler), without PCA transformation. Feature importance was ranked according to the mean absolute SHAP values, and beeswarm plots were generated to visualize the direction and relative magnitude of feature contributions. To ensure reproducibility, all random seeds were fixed (seed = 42).

Sensitivity analyses were conducted to evaluate the robustness of the classification results. Using the base model, which included CBT‐ and sleep‐derived indices as a reference, we repeated the machine‐learning analyses by (i) modifying the CBT data inclusion criterion to *R* ^2^ > 0.3 and (ii) additionally including hypnotic use as a covariate.

Analyses were performed in Python (v3.10.14) using scikit‐learn (v1.4.2), XGBoost (v1.7.6), LightGBM (v4.3.0), SHAP (v0.47.0), NumPy (v1.26.4), and pandas (v2.3.1).

### Ethical issues

Ethical guidelines were strictly followed throughout this study. All participants provided informed consent before participation. To ensure confidentiality, data were anonymized and analyzed in aggregate form. Participants were fully informed about the purpose of the study, data protection procedures, and their right to withdraw at any time without penalty. This study was conducted in accordance with the Declaration of Helsinki and approved by the Ethics Committee of the University of the Ryukyus (approval number: 23‐2158‐01‐00‐00).

## RESULTS

Table 1 summarizes the demographic and clinical characteristics of the two groups. Compared with the NC group, participants with BD were younger (30.8 ± 12.6 vs. 41.2 ± 10.1 years, *p* < 0.001) and had fewer years of education (13.0 ± 2.5 vs. 15.7 ± 1.9 years, *p* < 0.001). The proportion of married individuals was lower in the BD group (8.3%) than in the NC group (69.8%; *p* < 0.001). A family history of psychiatric disorders was more common in the BD group (56.5%) than in the NC group (9.3%; *p* < 0.001). Sex distribution and the proportion of individuals living alone were comparable between groups. Participants with BD had higher ISI scores (11.4 ± 5.7 vs. 5.3 ± 4.2, *p* < 0.001) and WHODAS 2.0 scores (22.0 ± 7.4 vs. 13.4 ± 2.3, *p* < 0.001). Regarding employment status, 95.3% of NC participants were employed, whereas in the BD group, 26.1% were employed, 26.1% were unemployed, 8.7% were on leave, 13.0% were receiving vocational support, and 26.1% were students. In the BD group, five participants (21.7%) had bipolar I disorder and 18 (78.3%) had bipolar II disorder. The mean age of onset was 21.0 ± 9.0 years. The mean illness duration was 9.8 ± 7.8 years. The mean HAMD‐17 total score was 6.3 ± 4.1, and the mean YMRS total score was 2.6 ± 3.8. At the time of assessment, most patients with BD were receiving pharmacotherapy (Table 1): mood stabilizers were prescribed in 17/23 (73.9%), antipsychotics in 18/23 (78.3%), hypnotics in 14/23 (60.9%), and antidepressants in 2/23 (8.7%). Details of the prescribed agents are provided in Table 1.

**Table 1:** Participant characteristics.

	Patients with bipolar disorder (*N* = 23) Mean ± SD or number (%)	Normal controls (*N* = 43) Mean ± SD or number (%)	*p*‐value
Demographic			
Age (years)^a^	30.8 ± 12.6	41.2 ± 10.1	<0.001*
Sex (male)^b^	8 (34.8)	17 (39.5)	0.705
Education (years)^a^	13.0 ± 2.5	15.7 ± 1.9	<0.001*
Married (yes)^c^	2 (8.3)	30 (69.8)	<0.001*
Living alone (yes)^c^	4 (16.7)	8 (18.6)	1.000
Family history of psychiatric disorders (yes)^c^	13 (56.5)	4 (9.3)	<0.001*
ISI total score^a^	11.4 ± 5.7	5.3 ± 4.2	<0.001*
WHODAS 2.0 total score^a^	22.0 ± 7.4	13.4 ± 2.3	<0.001*
Employment status			
Employed	6 (26.1)	41 (95.3)	‐
Unemployed	6 (26.1)	0 (0.0)	‐
On leave	2 (8.7)	0 (0.0)	‐
Vocational support	3 (13.0)	0 (0.0)	‐
Student	6 (26.1)	1 (2.3)	‐
Homemaker	0 (0.0)	1 (2.3)	‐
Background characteristics of bipolar disorder			
Bipolar disorder subtype (I/II), *n* (%)	5 (21.7)/18 (78.3)	‐	‐
Onset age of bipolar disorder (years)	21.0 ± 9.0	‐	‐
Illness duration (years)	9.8 ± 7.8		
HAMD‐17 total score	6.3 ± 4.1	‐	‐
YMRS total score	2.6 ± 3.8	‐	‐
Medication use			
Mood stabilizer use (≥1), *n* (%)	17 (73.9)	‐	‐
Antipsychotic use (≥1), *n* (%)	18 (78.3)	‐	‐
Hypnotic use (≥1), *n* (%)	14 (60.9)	‐	‐
Antidepressant use (≥1), *n* (%)	2 (8.7)	‐	‐
Mood stabilizers (specific agents)			
Lithium, *n* (%)	11 (47.8)	‐	‐
Valproate, *n* (%)	5 (21.7)	‐	‐
Lamotrigine, *n* (%)	2 (8.7)	‐	‐
Antipsychotics (specific agents)			
Lurasidone, *n* (%)	12 (52.2)	‐	‐
Aripiprazole, *n* (%)	4 (17.4)	‐	‐
Quetiapine, *n* (%)	4 (17.4)	‐	‐
Risperidone, *n* (%)	2 (8.7)	‐	‐
Hypnotics (specific agents)			
Lemborexant, *n* (%)	8 (34.8)	‐	‐
Eszopiclone, *n* (%)	3 (13.0)	‐	‐
Zolpidem, *n* (%)	2 (8.7)	‐	‐
Suvorexant, *n* (%)	1 (4.3)	‐	‐
Melatonin, *n* (%)	1 (4.3)	‐	‐
Flunitrazepam, *n* (%)	1 (4.3)	‐	‐
Brotizolam, *n* (%)	1 (4.3)	‐	‐
Ramelteon, *n* (%)	1 (4.3)	‐	‐

*Note: p* < 0.05 is considered statistically significant (*).

Abbreviations: HAMD‐17, 17‐item Hamilton Depression Rating Scale; ISI, Insomnia Severity Index; SD, standard deviation; WHODAS 2.0, World Health Organization Disability Assessment Schedule 2.0; YMRS, Young Mania Rating Scale.

^a^ Mann–Whitney *U* test.

^b^ Pearson's chi‐square test.

^c^ Fisher's exact test.

In the overall sample (*N* = 66), during the 3‐day CBT recording period, the mean wearable recording time was 61.5 ± 7.4 h, with 9.3 ± 6.4 h of missing data, corresponding to a missingness rate of 13.2% ± 9.1%. Overall model fit for CBT rhythm estimation was moderate (*R* ^2^ = 0.453 ± 0.136, range 0.16–0.72). Day‐to‐day within‐subject variability of the CBT nadir time across the 3‐day recording period, quantified as the within‐subject variance, was 1.99 ± 1.73 h^2^. The mean CBT nadir time was 5:33 ± 2:32 when calculated using all available CBT data and 5:36 ± 3:33 when restricted to segments with *R* ^2^ > 0.3, with a mean difference of +3 min (*R* ^2^ > 0.3 minus all data).

Table 2 presents the comparison of CBT nadir indicators, phase‐difference indicators, and actigraphy‐derived sleep indices between patients with BD and NC. The CBT nadir time was comparable between the two groups. Among the phase‐difference indicators, the midpoint–CBT nadir phase difference and the wake time–CBT nadir phase difference significantly differed between the BD and NC groups (83.5 ± 131.7 vs. 139.4 ± 128.0 min, *p* = 0.044; and 165.9 ± 133.9 vs. 70.4 ± 134.9 min, *p* = 0.004, respectively), whereas the bedtime–CBT nadir phase difference did not (332.9 ± 147.8 vs. 349.1 ± 126.2 min, *p* = 0.423). Regarding actigraphy‐derived sleep measures, patients with BD had longer total sleep time (459.6 ± 77.0 vs. 380.0 ± 49.5 min, *p* < 0.001), a later midpoint of sleep (4:22 ± 1:46 vs. 3:07 ± 0:51, *p* = 0.012), later wake‐up times (8:31 ± 1:40 vs. 6:36 ± 0:51, *p* < 0.001), greater variability in wake‐up time (106.8 ± 95.6 vs. 56.7 ± 29.3 min, *p* = 0.001), and greater variability in the midpoint of sleep (70.7 ± 44.2 vs. 51.1 ± 22.8 min, *p* = 0.034) than NCs. No notable group differences were observed for the remaining sleep parameters. After FDR correction for multiple comparisons among CBT‐ and actigraphy‐derived indices, the wake time–CBT nadir phase difference remained significant (FDR‐adjusted *p* = 0.013). Among actigraphy‐derived sleep measures, total sleep time, midpoint of sleep, wake time, and variability in wake‐up time also showed FDR‐adjusted *p*‐values below 0.05 (0.004, 0.031, 0.004, and 0.004, respectively).

**Table 2:** Comparison of core body temperature (CBT) nadir indicators, phase‐difference indicators, and actigraphy‐derived sleep indices between patients with bipolar disorder and normal controls.

	Patients with bipolar disorder (*N* = 23) Mean ± SD	Normal controls (*N* = 43) Mean ± SD	*p*‐value	FDR‐adjusted *p*‐value
CBT nadir indicator				
CBT nadir (o'clock)	5:45 ± 3:03	5:26 ± 2:15	1.000	1.000
Phase‐difference indicators				
Bedtime–CBT nadir phase difference (min)	332.9 ± 147.8	349.1 ± 126.2	0.423	0.611
Midpoint–CBT nadir phase difference (min)	83.5 ± 131.7	139.4 ± 128.0	0.044*	0.082
Wake time–CBT nadir phase difference (min)	165.9 ± 133.9	70.4 ± 134.9	0.004*	0.013*
Actigraphy‐derived sleep indices				
Total sleep time (min)	459.6 ± 77.0	380.0 ± 49.5	<0.001*	0.004*
Bedtime (o'clock)	0:12 ± 2:13	23:37 ± 1:03	0.642	0.759
Midpoint of sleep (o'clock)	4:22 ± 1:46	3:07 ± 0:51	0.012*	0.031*
Wake time (o'clock)	8:31 ± 1:40	6:36 ± 0:51	<0.001*	0.004*
Sleep efficiency (%)	89.2 ± 7.2	90.8 ± 5.1	0.711	0.770
Wake after sleep onset (min)	56.7 ± 38.0	39.0 ± 23.1	0.130	0.211
SD of bedtime (min)	80.7 ± 50.6	68.0 ± 29.7	0.586	0.759
SD of midpoint of sleep (min)	70.7 ± 44.2	51.1 ± 22.8	0.034*	0.074
SD of wake time (min)	106.8 ± 95.6	56.7 ± 29.3	0.001*	0.004*

*Note*: CBT nadir (o'clock) was estimated using cosinor analysis of distal body temperature recorded over 3 consecutive days. Actigraphy‐derived indices represent the 14‐day average values of sleep parameters. Phase‐difference indicators were calculated relative to the CBT nadir time as follows: bedtime–CBT nadir phase difference (min) = CBT nadir time − bedtime. Midpoint–CBT nadir phase difference (min) = CBT nadir time − sleep midpoint. Wake time–CBT nadir phase difference (min) = wake time − CBT nadir time. Group comparisons were performed using the Mann–Whitney *U* test. *p* < 0.05 was considered statistically significant (*).

Abbreviations: FDR, false discovery rate; SD, standard deviation.

Table 3 presents the results of multivariable linear regression analyses of CBT nadir and actigraphy‐derived sleep indices adjusted for diagnostic group and sociodemographic factors. After adjusting for age, sex, years of education, marital status, living arrangement, and employment status, diagnostic group (BD) was associated with a longer wake time–CBT nadir phase difference (*B* = 144.7 min, 95% CI, 26.8–262.6; *p* = 0.017), longer total sleep time (*B* = 114.2 min, 95% CI, 60.5–167.9; *p* < 0.001), and later wake time (*B* = 1.1 h, 95% CI, 0.1–2.1; *p* = 0.037). No clear independent association was observed between diagnostic group and CBT nadir time, bedtime, midpoint of sleep, sleep efficiency, wake after sleep onset, or other phase‐difference indices. Among sociodemographic variables, being married was associated with an earlier CBT nadir time (*B* = −1.8 h, 95% CI, −3.5 to −0.1; *p* = 0.034), earlier bedtime (*B* = −1.1 h, 95% CI, −2.1 to −0.1; *p* = 0.038), and earlier midpoint of sleep (*B* = −0.9 h, 95% CI, −1.7 to −0.1; *p* = 0.025). No clear associations were observed for age, sex, education, living alone, or employment status.

**Table 3:** Multivariable linear regression analyses of core body temperature (CBT) and sleep indices adjusted for diagnostic group and sociodemographic factors.

	CBT nadir (h)	Bedtime–CBT nadir phase difference (min)	Midpoint–CBT nadir phase difference (min)	Wake time–CBT nadir phase difference (min)	Total sleep time (min)	Bedtime (h)	Midpoint of sleep (h)	Wake time (h)	Sleep efficiency (%)	Wake after sleep onset (min)
Diagnostic group (BD = 1)	‐	‐	‐	144.7* (26.8–262.6)	114.2*(60.5–167.9)	‐	‐	1.1* (0.1–2.1)	‐	‐
Age (years)	‐	‐	‐	‐	‐	‐	‐	‐	‐	‐
Sex (female = 1)	‐	‐	‐	‐	‐	‐	‐	‐	‐	‐
Education (years)	‐	‐	‐	‐	‐	‐	‐	‐	‐	‐
Married (yes = 1)	−1.8* (−3.5 to −0.1)	‐	‐	‐	‐	−1.1* (−2.1 to −0.1)	−0.9* (−1.7 to −0.1)	‐	‐	‐
Living alone (yes = 1)	‐	‐	‐	‐	‐	‐	‐	‐	‐	‐
Employment (yes = 1)	‐	‐	‐	‐	‐	‐	‐	‐	‐	‐
Adjusted *R* ^2^	0.047	−0.061	0.011	0.101	0.264	0.084	0.269	0.429	−0.038	0.014

*Note*: Table entries show unstandardized regression coefficients (*B*) and 95% confidence intervals from multivariable linear regression models. Time‐of‐day variables, including CBT nadir, bedtime, midpoint of sleep, and wake time, are expressed in decimal h (1.0 = 60 min). Accordingly, regression coefficients reflect changes in h. *p* < 0.05 was considered statistically significant (*). “‐” indicates non‐significant associations (*p* ≥ 0.05) or values not shown for brevity.

Abbreviation: BD, bipolar disorder.

**Figure 2:**
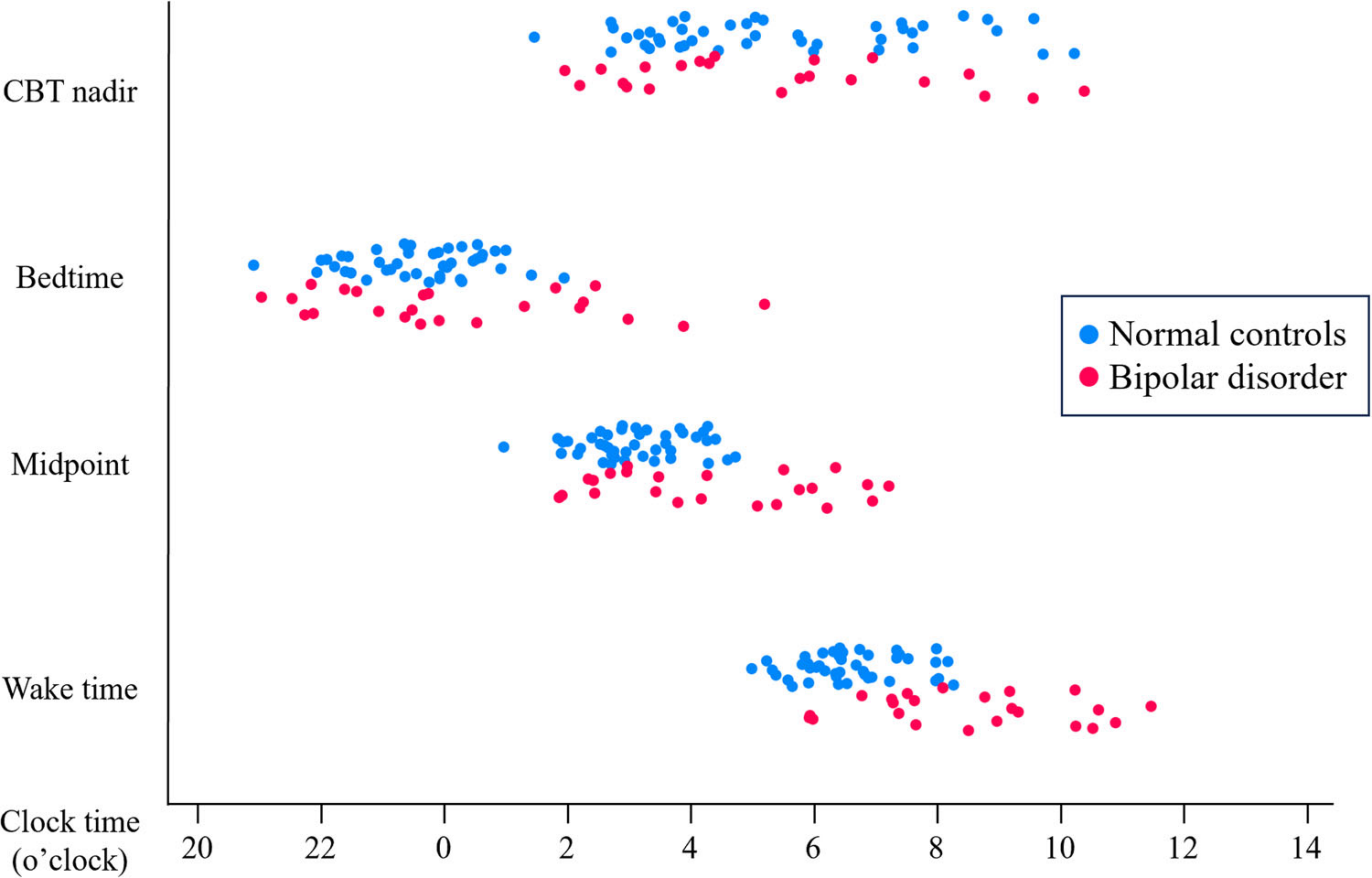
Clock‐time distribution of the core body temperature (CBT) nadir and sleep–wake timing (bedtime, sleep midpoint, and wake time) in normal controls and patients with bipolar disorder.

Figure 2 displays the distributions of CBT nadir, bedtime, sleep midpoint, and wake time (clock times) in the BD and NC groups.

**Figure 3:**
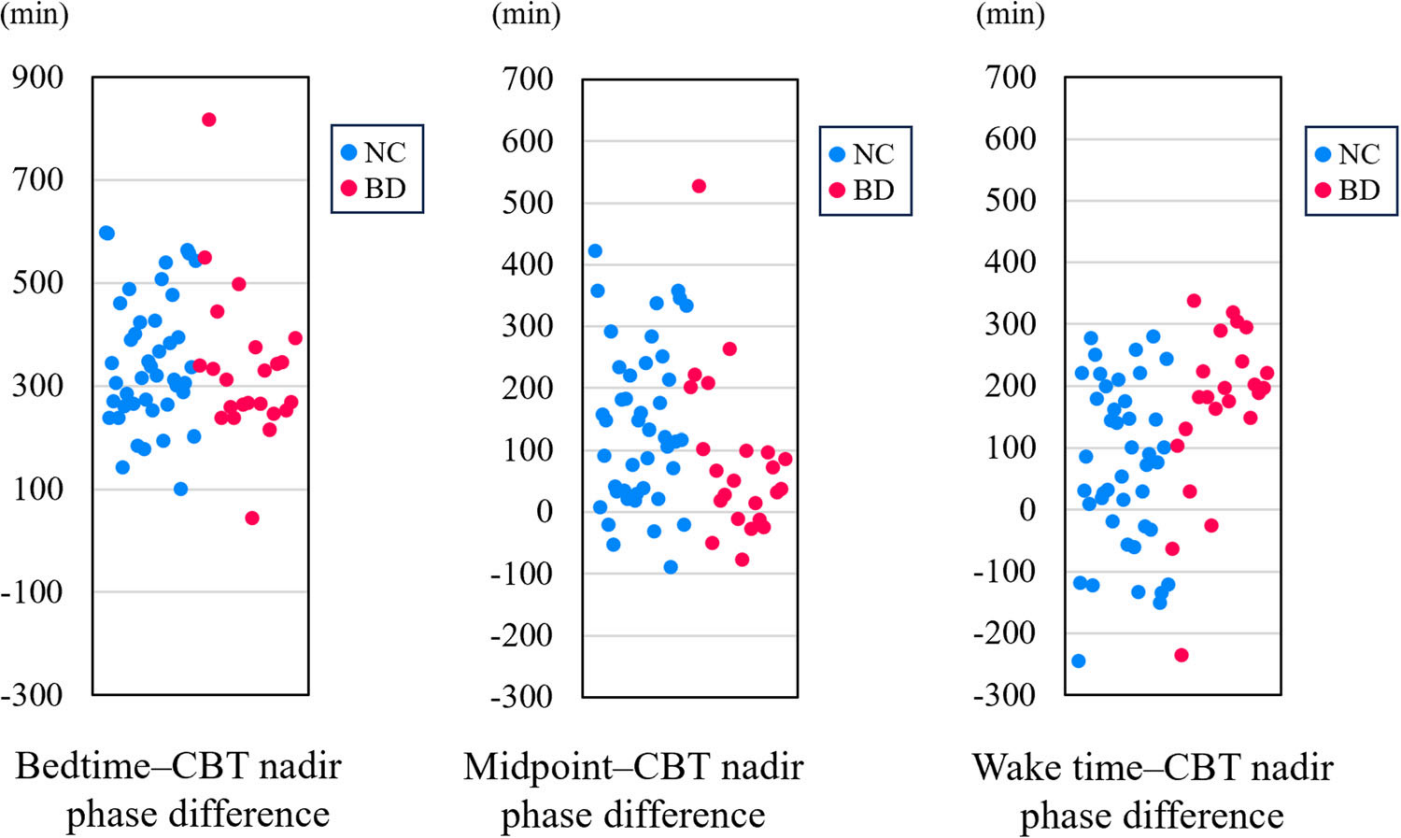
Scatter plots of the bedtime–, midpoint–, and wake time–CBT nadir phase differences in normal controls (NCs) and patients with bipolar disorder (BD). *Note*: In this study, phase‐difference indicators were calculated relative to the CBT nadir time as follows: bedtime–CBT nadir phase difference (min) = CBT nadir time − bedtime. Midpoint–CBT nadir phase difference (min) = CBT nadir time − sleep midpoint. Wake time–CBT nadir phase difference (min) = wake time − CBT nadir time.

Figure 3 shows scatter plots of the bedtime–CBT nadir, midpoint–CBT nadir, and wake time–CBT nadir phase differences in the BD and NC groups.

Table 4 summarizes the performance of the base model (sleep + CBT) and the extended model (sleep + CBT + demographic variables) evaluated using repeated nested cross‐validation at the MaxF1 operating point (25 outer folds). For the base model, the mean ROC–AUC was 0.771 ± 0.162 (95% CI, 0.586–0.905), and accuracy was 0.713 ± 0.059 (95% CI, 0.643–0.793). Sensitivity and specificity at the MaxF1 operating point were 0.750 ± 0.306 and 0.714 ± 0.175, respectively. The F1 score was 0.617 ± 0.132 (95% CI, 0.420–0.742). For the extended model, the mean ROC–AUC was 0.930 ± 0.050 (95% CI, 0.877–0.995), and accuracy was 0.751 ± 0.046 (95% CI, 0.703–0.800). Sensitivity and specificity at the MaxF1 operating point were 0.633 ± 0.280 and 0.829 ± 0.120, respectively. The F1 score was 0.611 ± 0.134 (95% CI, 0.417–0.742).

**Table 4:** Nested cross‐validation performance of the base and extended models at the MaxF1 operating point.

Metrics	Base model (sleep + CBT)	Extended model (+demographics)
ROC–AUC (mean ± SD)	0.771 ± 0.162 (95% CI, 0.586–0.905)	0.930 ± 0.050 (95% CI, 0.877–0.995)
Accuracy	0.713 ± 0.059 (0.643–0.793)	0.751 ± 0.046 (0.703–0.800)
Sensitivity	0.750 ± 0.306 (0.300–1.000)	0.633 ± 0.280 (0.275–0.975)
Specificity	0.714 ± 0.175 (0.571–0.971)	0.829 ± 0.120 (0.714–0.986)
F1 score	0.617 ± 0.132 (0.420–0.742)	0.611 ± 0.134 (0.417–0.742)

*Note*: Values represent the distribution across outer folds in repeated nested cross‐validation (outer: stratified fivefold repeated 5 times; total outer folds = 25). Metrics are reported at the MaxF1 operating point as mean ± standard deviation and 95% confidence intervals derived from the outer‐fold distribution. The MaxF1 threshold was determined using out‐of‐fold predicted probabilities within the training data.

Abbreviations: CBT, core body temperature; CI, confidence interval; F1, harmonic mean of precision and recall; ROC–AUC, area under the receiver operating characteristic curve; SD, standard deviation.

As a supplementary assessment, performance on the independent holdout test set (*n* = 14) was evaluated (Table S1). In this small holdout set, the base and extended models yielded ROC–AUC values of 0.933 and 1.000, respectively. Additional threshold‐dependent and calibration metrics are also summarized in Table S1.

**Figure 4:**
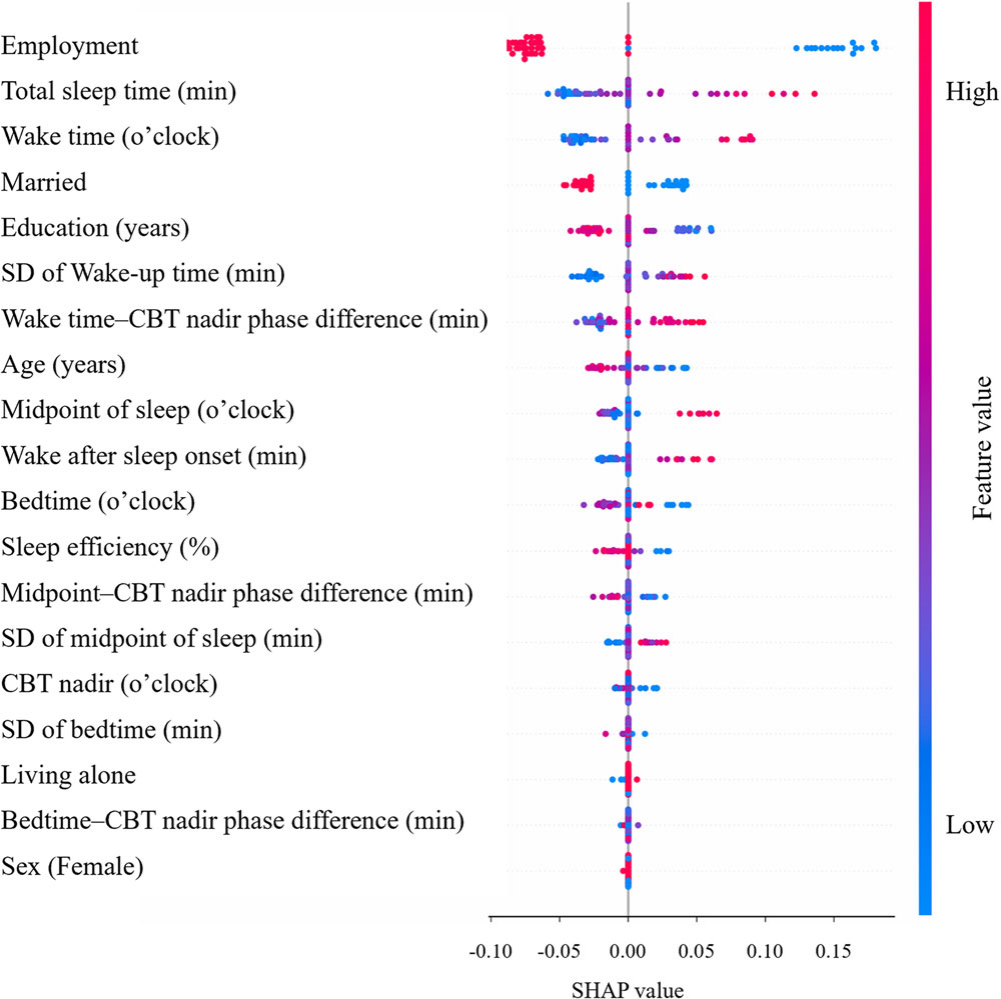
SHapley Additive exPlanations (SHAP) summary plot of relative feature contributions to the final extended model output. *Note*: SHAP values represent relative feature contributions of each feature to the model output (feature importance) and should not be interpreted as a clinical effect size. Each point corresponds to a single observation, with color indicating the feature value (blue = low, red = high). Positive SHAP values indicate a higher probability of classification as bipolar disorder (BD), whereas negative SHAP values indicate a higher probability of classification as normal control (NC). CBT, core body temperature.

Figure 4 presents the SHAP values, which represent the relative contributions of each feature to the model output. The figure visualizes the impact of CBT nadir indicators, phase‐difference indicators, actigraphy‐derived sleep indices, and sociodemographic variables on the output of the final extended model. The seven most influential features were employment status, total sleep time, wake time, marital status, years of education, variability in wake‐up time, and the wake time–CBT nadir phase difference.

To illustrate potential sensitivity–specificity trade‐offs, we evaluated three predefined operating points within the nested cross‐validation framework (Table S2). At the F1‐maximizing threshold, sensitivity and specificity were 0.75 ± 0.31 and 0.71 ± 0.18, respectively (accuracy 0.71 ± 0.06; AUC 0.77 ± 0.16). The sensitivity‐prioritized operating point yielded a similar mean sensitivity (0.75 ± 0.31) with a modest reduction in specificity (0.68 ± 0.06) on the outer test folds (accuracy 0.69 ± 0.13; AUC 0.77 ± 0.16). In contrast, the specificity‐prioritized operating point increased specificity to 0.91 ± 0.13 at the expense of sensitivity (0.50 ± 0.21) (accuracy 0.77 ± 0.11; AUC 0.77 ± 0.16).

Sensitivity analyses yielded results broadly consistent with those of the base model (Table S3). In nested cross‐validation, the mean AUC increased from 0.77 ± 0.16 in the base model to 0.83 ± 0.14 when applying the alternative CBT inclusion criterion (*R* ^2^ > 0.3) and further to 0.86 ± 0.10 when hypnotic use was included as a covariate. On the independent holdout test set, overall classification performance remained comparable across models. Accuracy, balanced accuracy, sensitivity, and specificity were unchanged across analysis conditions (accuracy = 0.857; sensitivity = 0.800; and specificity = 0.889), while small variations were observed in ROC–AUC, average precision, and Brier score.

## DISCUSSION

To our knowledge, this study is the first to integrate wearable‐derived CBT data with actigraphy‐derived sleep indices within a machine‐learning framework to differentiate patients with BD from NC. A previous study using 90‐day actigraphy of motor activity reported 88% classification accuracy (sensitivity, 85%; specificity, 91%).^11^ Another study using 21‐day actigraphy reported that combining activity and sleep variability parameters enabled the discrimination between BD and NC, with classification rates of 80% for BD and 75% for NC, and an AUC of 0.86.^12^ Whereas these prior investigations primarily examined classification based on actigraphy‐derived measures, the present study explored an approach integrating CBT indices in addition to sleep measures. In repeated nested cross‐validation, the base model (sleep + CBT) achieved an ROC–AUC of 0.771 ± 0.162 (95% CI, 0.586–0.905) and an accuracy of 0.713 ± 0.059 (Table 4). However, the present study was designed as an exploratory proof‐of‐concept analysis incorporating CBT indices, and important constraints remain. However, the present study was designed as an exploratory proof‐of‐concept analysis incorporating CBT indices, and several important constraints remain. In particular, employment status was imbalanced between the BD and NC groups (Table 1), and employment‐related variables were also identified as potentially influential features in the SHAP‐based interpretation of the extended model (Figure 4). Therefore, the findings observed in this study may not necessarily represent disorder‐specific biomarkers but partly reflect lifestyle differences related to the presence or absence of social roles, particularly employment—that is, social zeitgebers. In addition, the relatively short data‐collection period, particularly the 3‐day CBT assessment, may have limited the stability of the CBT‐related indices. Accordingly, the present findings should be interpreted cautiously as hypothesis‐generating observations within the context of these limitations.

From the perspective of CBT‐related indices, findings from both conventional statistical models and machine‐learning–based interpretability analyses suggested that, within the present dataset, the wake time–CBT nadir phase difference may be associated with BD–NC discrimination. In the group comparison, the wake time–CBT nadir phase difference showed an FDR‐adjusted *p*‐value below 0.05 (Table 2). In covariate‐adjusted multivariable linear regression analyses, the wake time–CBT nadir phase difference was also associated with diagnostic group after adjustment for sociodemographic factors (Table 3). Moreover, in the extended model incorporating sociodemographic variables, SHAP‐based interpretability analyses ranked this phase‐difference indicator among the higher‐contributing features (Figure 4). Notably, CBT nadir timing itself did not show a clear difference between BD and NC, whereas wake time was delayed in BD (Table 3), suggesting that the enlarged phase difference may primarily reflect differences in wake timing rather than shifts in the CBT nadir. Previous studies using rectal CBT recordings have shown that, in patients with DSPS, the wake time–CBT nadir phase difference is larger than that in NC.^31^ Another study reported that, in DSPS, the bedtime–CBT nadir phase difference did not differ from that in NC, whereas the wake time–CBT nadir phase difference was enlarged.^21^ These findings in DSPS are broadly consistent with the results observed in patients with BD. Although the direct relationship between BD and CBT has not been clarified, several studies have reported an association between BD and a delayed sleep–wake rhythm.^22^ Although the present study is small and exploratory, it offers preliminary observations suggesting that an enlarged wake time–CBT nadir phase difference may be related to BD.

In the present study, total sleep time was suggested to be associated with BD–NC discrimination based on both the covariate‐adjusted multivariable linear regression analyses (Table 3) and the SHAP‐based interpretability results of the extended machine‐learning model incorporating sociodemographic factors (Figure 4). Meta‐analytic evidence has reported that individuals with remitted BD exhibit longer total sleep time than NCs.^32^ Another systematic review and meta‐analysis likewise indicated prolonged total sleep time in remitted BD.^33^ Collectively, these observations are consistent with our findings and suggest that total sleep time may capture a characteristic feature of BD. Although participants with BD were enrolled in a remitted state (HAMD‐17 total score 6.3 ± 4.1; YMRS total score 2.6 ± 3.8), the mean ISI total score was 11.4 ± 5.7, suggesting that residual insomnia symptoms may have persisted in a subset of participants. In this context, total sleep time may represent a clinically relevant indicator characterizing BD, even during remission.

The primary aim of this study was to explore, from the perspective of discriminating BD from healthy controls, the technical and methodological feasibility of incorporating wearable‐derived circadian measures, including CBT‐related indices, into future diagnostic‐support frameworks. From a clinical standpoint, BD is not infrequently misdiagnosed at initial presentation, and insufficient history‐taking has been identified as a major contributor to such misdiagnosis.^34^ In this context, objective indicators of sleep, activity, and circadian rhythms may provide complementary information to symptom‐based assessments. However, a more central clinical challenge in BD lies in differential diagnosis, as symptom overlap with other psychiatric conditions and secondary factors often leads to diagnostic uncertainty. Differentiating bipolar depression from unipolar depression remains particularly challenging, and a substantial proportion of individuals with BD are initially diagnosed with major depressive disorder.^35^ In addition, borderline personality features and the effects of substance use may overlap with (hypo)manic symptoms and contribute to diagnostic confusion, while in pediatric populations, attention‐deficit/hyperactivity disorder symptoms may be misinterpreted as indicative of BD.^36^ To illustrate sensitivity–specificity trade‐offs, we additionally presented high‐sensitivity (HighSens) and high‐specificity (HighSpec) operating points on an exploratory basis (Table S2). Future studies should extend these preliminary findings to designs with greater clinical utility, such as comparisons including disorders that commonly pose differential diagnostic challenges. In such settings, sensitivity‐prioritized thresholds may be useful when minimizing missed detection of BD is a priority, whereas specificity‐prioritized thresholds may be preferable when overdiagnosis due to the coexistence of other disorders is a concern.

This study has some limitations. First, the sample size of the present study was relatively small. To confirm the generalizability of the findings, larger studies in cohorts with more balanced background characteristics and matched on key sociodemographic factors are warranted. Although we attempted to mitigate potential confounding by conducting multivariable linear regression analyses with relevant covariates, constructing an extended machine‐learning model incorporating sociodemographic variables, and performing sensitivity analyses including hypnotic use, residual confounding cannot be fully excluded. In particular, the potential effects of medications such as hypnotics and mood stabilizers on sleep and CBT rhythms should be considered. Second, to assess whether the primary findings could be driven by CBT measurement artifacts, we conducted a sensitivity analysis stratified by the cosinor goodness of fit (*R* ^2^). The primary findings were unlikely to be substantially explained by lower fit CBT segments. However, the recording period was short (3 days), and the stability of the estimated nadir timing cannot be fully assured. In addition, the *R* ^2^ value used as a cosinor fit index merely reflects how well a rhythmic pattern is captured by the statistical model and does not independently validate external validity beyond model fit. Therefore, the CBT‐related results should be interpreted with caution. Third, the present analyses were conducted within a framework of discriminating BD from healthy controls. Therefore, although the machine‐learning analyses suggested features associated with BD, the study was not designed to distinguish BD from major depressive disorder among patients presenting with depressive symptoms. Future studies are warranted in clinically enriched cohorts that include key differential diagnostic comparators.

In conclusion, this small‐sample, exploratory proof‐of‐concept study suggests that a classification approach combining wearable‐derived CBT indices and actigraphy‐based sleep parameters may discriminate BD from NC. Across covariate‐adjusted analyses and model‐interpretability assessments incorporating sociodemographic factors, the wake time–CBT nadir phase difference, wake time, and total sleep time emerged as indices potentially associated with BD–NC discrimination. However, given the modest sample size, the potential for residual confounding due to sociodemographic imbalance, and the study design focusing on discrimination from healthy controls, the present findings should be considered preliminary. Future studies are warranted in larger cohorts with more balanced and matched background characteristics, as well as in clinically enriched samples that include disorders requiring differential diagnosis, to further evaluate the clinical utility of this approach.

## AUTHOR CONTRIBUTIONS

Kazuhiro Kurihara wrote the original draft, methodology, and formal analysis. Ayano Shiroma contributed to data collection, writing, review and editing, methodology, and formal analysis. Masaki Kamata contributed to writing, review, editing, methodology, and formal analysis. Naruyo Yoshimoto contributed to data collection, writing, review, and editing. Ayano Saso contributed to data collection, writing, review, and editing. Akiyoshi Shimura contributed to data curation, formal analysis, writing, review, and editing. Yukiko Imai contributed to device provision, data preprocessing, writing, review, and editing. Tadashi Miyahara contributed to device provision, data preprocessing, writing, review, and editing. Yoshikazu Takaesu contributed to writing, review, editing, formal analysis, investigation, project administration, funding acquisition, and overall supervision.

## CONFLICT OF INTEREST STATEMENT

Yoshikazu Takaesu received lecture fees from Takeda Pharmaceutical, Otsuka Pharmaceutical, Daiichi Sankyo Company, Shionogi, Mochida Pharmaceutical, Lundbeck Japan, Eisai, MSD, and Viatris Pharmaceuticals outside of the submitted work. Akiyoshi Shimura received lecture fees from Eisai Co., Ltd. and MSD K.K. The other authors declare no competing financial interests or personal relationships that could influence the work reported in this study.

## ETHICS APPROVAL STATEMENT

This study was approved by the Ethics Committee of the University of the Ryukyus (approval number: 23‐2158‐01‐00‐00).

## PATIENT CONSENT STATEMENT

All participants provided written informed consent before participating in the study.

## CLINICAL TRIAL REGISTRATION

Not applicable.

## Supporting information

Supporting File 1.

## Data Availability

The data that support the findings of this study are available on request from the corresponding author. The data are not publicly available due to privacy or ethical restrictions.
