# Supplementary material for: Machine‐learning classification of bipolar disorder incorporating wearable‐derived core body temperature and actigraphy‐derived sleep indices
Source: PCN Rep. 2026 Jun 8;5(2):e70359. doi: 10.1002/pcn5.70359 (PMC13244259; doi:10.1002/pcn5.70359)
Supplement: Supplementary file 1 — Supporting File 1. [file PCN5-5-e70359-s001.docx]

**Supplementary Table S1.** Performance on independent holdout test set

| Metrics | Base model (Sleep + CBT) | Extended model (+ Demographics) |
| --- | --- | --- |
| ROC-AUC | 0.933 | 1.000 |
| Average Precision | 0.925 | 1.000 |
| Brier Score | 0.147 | 0.085 |
| Accuracy | 0.857 | 1.000 |
| Sensitivity | 0.800 | 1.000 |
| Specificity | 0.889 | 1.000 |
| F1 Score | 0.800 | 1.000 |

**Abbreviations:** ROC-AUC, Area Under the Receiver Operating Characteristic Curve; F1, harmonic mean of precision and recall.

**Note**: Performance was evaluated on the independent holdout test set (n = 14). Threshold-dependent metrics (accuracy, sensitivity, specificity, and F1 score) were calculated using the MaxF1 threshold determined from the training data.

**Supplementary Table S2.** Nested cross-validation performance of the Base model at predefined operating points

| Operating point | Sensitivity (mean ± SD) | Specificity (mean ± SD) | Accuracy (mean ± SD) | F1 score (mean ± SD) | AUC (mean ± SD) |
| --- | --- | --- | --- | --- | --- |
| F1-max | 0.75 ± 0.31 | 0.71 ± 0.18 | 0.71 ± 0.06 | 0.62 ± 0.13 | 0.77 ± 0.16 |
| Sensitivity-prioritized | 0.75 ± 0.31 | 0.68 ± 0.06 | 0.69 ± 0.13 | 0.61 ± 0.19 | 0.77 ± 0.16 |
| Specificity-prioritized | 0.50 ± 0.21 | 0.91 ± 0.13 | 0.77 ± 0.11 | 0.59 ± 0.18 | 0.77 ± 0.16 |

**Abbreviations:** F1, harmonic mean of precision and recall; AUC, Area Under the Receiver Operating Characteristic Curve; SD, standard deviation.

**Note:** The primary operating point was defined as the threshold maximizing the F1 score (F1-max). Sensitivity- and specificity-prioritized operating points were defined using out-of-fold predicted probabilities within the training data, targeting sensitivity ≥ 0.90 and specificity ≥ 0.90, respectively. Among eligible thresholds, the threshold maximizing the complementary metric was selected. Performance was evaluated on the held-out outer folds and summarized as mean ± SD across folds.

**Supplementary Table S3.** Nested cross-validation performance of the Base model at predefined operating points

| Analysis condition | Nested CV AUC  (mean ± SD) | Holdout ROC-AUC | Holdout AP | Holdout Brier | Holdout Accuracy | Holdout Balanced Acc | Holdout Sens | Holdout Spec |
| --- | --- | --- | --- | --- | --- | --- | --- | --- |
| Base model | 0.771 ± 0.162 | 0.933 | 0.925 | 0.147 | 0.857 | 0.844 | 0.800 | 0.889 |
| (i) CBT inclusion criterion: R² > 0.3 | 0.830 ± 0.142 | 0.911 | 0.911 | 0.150 | 0.857 | 0.844 | 0.800 | 0.889 |
| (ii) + Hypnotic use as an additional covariate | 0.860 ± 0.096 | 0.978 | 0.967 | 0.102 | 0.857 | 0.844 | 0.800 | 0.889 |

**Abbreviations:** AP, average precision; ROC-AUC, Area Under the Receiver Operating Characteristic Curve; CBT, core body temperature; CV, cross-validation; ROC-AUC, area under the receiver operating characteristic curve; SD, standard deviation; F1, harmonic mean of precision and recall.

**Note:** Sensitivity analyses were performed to assess robustness of the primary classification results by (i) applying an alternative CBT data inclusion criterion (R² > 0.3) and (ii) additionally including hypnotic use as an input feature. Nested CV metrics represent mean ± SD across outer folds. Holdout metrics were evaluated on the independent test set (n = 14) using the F1-maximizing threshold.
